# The P3 Reflects Awareness and Can Be Modulated by Confidence

**DOI:** 10.3389/fnins.2019.00510

**Published:** 2019-05-17

**Authors:** Muwang Ye, Yong Lyu, Ben Sclodnick, Hong-Jin Sun

**Affiliations:** ^1^Academy of Psychology and Behavior, Tianjin Normal University, Tianjin, China; ^2^Department of Psychology, Neuroscience & Behavior, McMaster University, Hamilton, ON, Canada

**Keywords:** neural correlate of consciousness, confidence, P3, visual awareness negativity, event-related potential

## Abstract

An important question in neural correlate of consciousness (NCC) studies is whether event-related potential (ERP) component P3 reflects visual awareness or the confidence with which one reports a visual experience. In the present study, participants detected visual stimuli presented at threshold-level contrast, then rated their subjective confidence with respect to their response on a four-point scale (very confident, quite confident, slightly confident, and not confident at all). Because awareness responses in trials with rating of “not confident at all” were likely noise, we analyzed the data excluding those trials. The ERP results revealed a significant positive difference in P3 amplitude between “aware” and “unaware” trials. P3 amplitude was more positive in aware trials compared to unaware trials. Importantly, this pattern was observed for trials with combined confidence ratings of “very confident” and “quite confident,” and for trials with confidence ratings of “slightly confident,” suggesting that awareness alone can modulate P3. A significant interaction between awareness and confidence is reported, suggesting that confidence influences P3 as well. In addition, ERP results revealed that visual awareness negativity (VAN) was observed over posterior temporal and occipital electrodes and largely not influenced by confidence. This result indicated that VAN is an early neural correlate of visual awareness.

## Introduction

The neural correlate of consciousness (NCC) is defined as “minimum neural mechanism jointly sufficient for any one specific conscious experience” (Koch et al., 2016, p. 307). Searching for the NCC has become a central endeavor in neuroscience. Using electro-encephalography (EEG), researchers can empirically capture the unfolding of neural events that correspond to subjective conscious experience. Through precise timing of electrophysiological events in response to stimuli, ERP recording is an ideal method for identifying NCC.

Over the past 20 years, most ERP studies on NCC focused on visual processing. In particular, researchers recorded ERP during the presentation of a threshold-level visual stimulus. The presentation of such weak stimuli ensured that on some trials participants were aware of the stimulus, and on other trials they were not. Researchers then contrasted brain ERP’s from “aware” trials with “unaware” trials for the same stimulus intensity (contrastive experimental design) (Baars, 1989), allowing them to make inferences about NCC (Rutiku and Bachmann, 2017).

By using such contrastive experimental designs, two candidate electrophysiological NCCs have emerged: visual awareness negativity (VAN) and late positivity (LP). VAN is the negative amplitude difference between aware and unaware conditions (Koivisto and Revonsuo, 2010; Railo et al., 2011). It typically appears around 200 ms (N1–N2 latency range or N200) in posterior temporal and occipital electrodes (Railo et al., 2011; Koivisto et al., 2016, 2017; Koivisto and Grassini, 2016; Eklund and Wiens, 2018). LP is the positive amplitude difference between aware and unaware condition in the P3 time window (Koivisto and Revonsuo, 2010; Railo et al., 2011). LP is typically found at parietal electrodes (Dehaene and Changeux, 2011; Dehaene et al., 2014; Rutiku et al., 2015; Naccache et al., 2016).

However, it has been argued that the P3 may reflect variations in participants’ confidence with respect to their response (Eimer and Mazza, 2005). Because NCC studies typically present stimuli at near-threshold level, participants are often not confident about whether they detected the stimulus on any given trial. If the participants adopted a conservative response criterion, the difference between aware and unaware trials might be associated with the participants’ confidence, even in instances when they do not need to report their level of confidence (Salti et al., 2012).

Eimer and Mazza (2005) adopted a change detection task to investigate whether or not P3 reflected confidence with respect to being aware versus being unaware of the change. In their study, the participants first monitored displays containing four faces, then detected a face identity change across successive displays. Finally, participants rated their confidence on a three-point scale (100% confident, 50% confident, and 0% confident). Eimer and Mazza (2005) found that the P3 amplitude of high confidence was more positive in aware trials compared to unaware trials, but no significant P3 differences between aware trials and unaware trials were found when the participants’ confidence was low. Eimer and Mazza (2005) thus suggested that the positive amplitude difference between aware and unaware condition in the P3 time window (LP) likely represented participant’s confidence based on an observed relation between P3 amplitude and participants’ confidence (in their responses).

Despite the findings reported by Eimer and Mazza (2005), there is mounting evidence that P3 indeed reflects visual awareness rather than confidence (Lamy et al., 2009; Salti et al., 2012). Salti et al. (2012) examined whether P3 correlated with visual awareness when confidence is controlled for. In their study, participants performed a forced-choice location detection task and then reported their conscious perception of the target on a three-point scale (certain I saw, unsure, and certain I did not see). This three-point scale essentially indexed participants’ confidence. They found that P3 amplitude was more positive in aware trials compared with unaware trials when participants reported high confidence. Consequently Salti et al. (2012) argued that the P3 reflected visual awareness. In addition, they argued that Eimer and Mazza (2005) could not provide sufficient evidence to support that P3 reflected the participants’ confidence. In particular, Eimer and Mazza (2005) combined 0% confident and 50% confident trials into a low confidence condition. When the participants’ confidence rating was 0% confidence, it is reasonable to infer that their response was pure guess (Lamy et al., 2009; Salti et al., 2012). Therefore awareness level could be similar between the aware and unaware trials in low confident trials. When comparing ERP data for “aware” vs. “unaware” trials, trials with low confidence ratings would unnecessarily introduce noise. This could explain why Eimer and Mazza (2005) did not find effects of subjective awareness on P3 amplitude in low confident trials.

Given the debate in the literature (Eimer and Mazza, 2005; Lamy et al., 2009; Salti et al., 2012), the aim of present study was to reexamine whether the P3 can be modulated by visual awareness and/or confidence. We speculated that results of previous studies (Eimer and Mazza, 2005) could be contaminated by the results from 0% confidence trials. In particular, the effect of confidence (P3 was modulated by confidence) could in theory result from greater noise in the data from 0% confidence trials. We thus minimized this potential confound by increasing the sensitivity of our confidence scale and separately analyze data with and without trials with 0% confidence rating.

We used a forced-choice detection task, modified after that of Koivisto et al. (2016). Following stimulus presentation, participants first indicated whether they had seen the stimulus – by pressing one of two designated keys – then rated their confidence in their response according to the confidence scale modified after Sandberg et al. (2010). The confidence rating utilized a four-point confidence rating scale: (1) very confident, (2) quite confident, (3) slightly confident, and (4) not confident at all. The four-point scale would offer us with multiple levels of confidence rating to examine the possible contribution of confidence. By excluding results from “no confidence” trials, our measures should be more sensitive to amplitude of P3 in relation to reports of awareness and confidence thereof. In addition, with ERP data, we were also able to reexamine earlier VAN and its relation with awareness and confidence. VAN was traditionally considered to be related to awareness only (Koivisto et al., 2016; Koivisto and Grassini, 2016).

## Materials and Methods

### Participants

Thirty-one right-handed undergraduate students participated in this study. However, 15 participants were excluded. Among them, 4 participants reported awareness in less than 25% of the critical trials or more than 75% of the critical trials and 11 participants did not have enough (>20) critical trials for each awareness/confidence combination used in the data analysis (see section “EEG Recording and Data Analysis” below). The final sample consisted of 16 participants (five males, aged between 19 and 27 years, *M* = 22.25, *SD* = 2.59). All participants had normal or corrected to normal eyesight; none of them had history of neurological disease or brain injuries. All participants gave written informed consent prior to the study. All procedures were approved by the Ethics Committee in Academy of Psychology and Behavior, Tianjin Normal University.

### Stimuli and Apparatus

Stimuli were controlled with E-prime software on a 1024 × 768 resolution monitor with 60 Hz refresh rate. The luminance of the gray background was 22 cd/m^2^. The critical stimulus was a low contrast sinusoidal Gabor patch (4.24 degree in diameter, Michelson contrast = 0.05), tilted 45 degree to the left.

### Procedure

**FIGURE 1:**
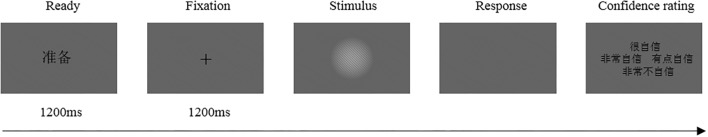
Each trial started with the presentation of a Chinese word “
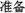
” (i.e. “READY”) followed by a black fixation cross. Then, the stimulus (or a blank screen in catch trials) was presented. After the stimulus (or blank), the participants indicated whether they had seen the stimulus then rated their subjective confidence with respect to their response on a four-point scale. The confidence rating utilized a four-point confidence rating scale: (1) “
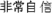
” (i.e. “very confident”), (2) “
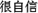
” (i.e. “quite confident”), (3) “
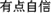
” (i.e. “slightly confident”), (4) “
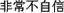
” (i.e. “not confident at all”).

Each trial started with the presentation of a Chinese word “
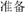
” (i.e., “READY”) at the center of the screen for 1200 ms. Next, a black fixation cross was presented for 1200 ms, followed by the stimulus (or a blank screen in catch trials). The fixation cross and stimulus were presented in the center of the screen. Stimulus duration was calibrated to each participant’s perception threshold (see below). After the stimulus (or blank), the participants indicated whether they had seen the stimulus by pressing one of two designated buttons using the same hand. Finally, a prompt appeared on the screen asking participants to rate their subjective confidence with respect to their response on a four-point scale (Sandberg et al., 2010; Figure 1).

A total of 720 trials were conducted in six blocks of stimuli, separated by brief resting periods. Each block consisted of 80 critical trials, 20 catch trials, and 20 control trials. The catch trials contained no stimuli. In control trials, a higher stimulus contrast (Michelson contrast: 0.2) was used than in the critical trials.

In pre-experimental calibration phase, the stimulus presentation was identical to that in the main experiment. The participants were instructed to indicate whether they had seen the stimulus in each trial, but they were not instructed to rate their subjective confidence. In the first calibration block, the duration of the critical stimuli was two frames. Each calibration block included 20 critical trials, 5 catch trials, and 5 control trials. In next block, the number of screen refreshes was increased or decreased by one frame according to the participants’ performance. If the participants reported awareness in less than 25% or more than 75% of the critical trials, the number of refresh frames was increased or decreased with one frame correspondingly. When the appropriate stimulus duration was found, the same calibration block was repeated once again to make sure that performance remained stable.

### EEG Recording and Data Analysis

The EEG was recorded from 64-channel Ag/AgCl electrode cap (NeuroScan, Melbourne, VIC, Australia) according to the 10–20 system. Vertical EOG recording electrodes were positioned above and below the left eye, and horizontal EOG recording electrodes were positioned 1.5 cm from the outer canthus of each eye. The reference electrode was placed on the nose. The ground electrode was placed in front of Fz. The EEG and EOG signals were amplified with a band pass of 0.05–400 Hz and sampled at 1000 Hz. The impedance was kept below 5 kΩ.

Electro-encephalography data were analyzed offline with Curry 7. Offline correction of eye movement artifact was performed. To exclude trials contaminated by artifacts, trials with voltages exceeding ± 80 μV at any electrodes were discarded. The signals were averaged offline over 800 ms periods, and an additional 100 ms was recorded prior to the probe onset to allow for baseline correction. The data were filtered with 0.1 Hz high pass and 30 Hz low pass filters.

In terms of confidence ratings, participants rarely chose “very confident.” For this reason the data from “very confident” trials and “quite confident” trials were combined into a high confidence dataset to increase statistical power. Additionally, the data from “not confident at all” were eliminated from ERP analyzed. Therefore ERPs for four types of critical trials were obtained (trial number statistics provided in bracket): aware/high-confidence (*M* = 71, *SD* = 37.1), aware/slight-confidence (*M* = 73, *SD* = 29.94), unaware/high-confidence (*M* = 160, *SD* = 82.02), and unaware/slight-confidence (*M* = 65, *SD* = 33.96).

Based on previous similar studies (Koivisto et al., 2006, 2016; Lamy et al., 2009; Salti et al., 2012) and inspection of the grand-averaged ERPs, the ERP components and their time epochs were partitioned as following: N1: 200–300 ms; N2: 325–375 ms; and P3: 500–600 ms. The measured data of each component were analyzed using SPSS 22. Repeated-measures analysis of variance (ANOVA) was performed with the following factors: Awareness (2: aware, unaware), Confidence (2: high, slight), Area [3: parietal (P3, P4), posterior temporal (P7, P8), occipital (O1, O2)], and Hemisphere (2: left vs. right). The Greenhouse-Geisser correction was applied when the sphericity assumption was violated.

## Results

### Behavioral Results

Participants (*n* = 16) reported awareness in 41.02% (*SD* = 13.37) of the critical trials; this level of awareness was appropriate, as our goal was to obtain about 50% awareness. The proportions of response for each awareness/confidence combination are presented in Table 1.

**Table 1:** The proportion of trials at each confidence rating level for the critical low-contrast stimulus as a function of the awareness (*M* ± *SD*).

	Critical:	Critical:
	aware (%)	unaware (%)
very confident	3.96 (3.48)	11.16 (13.16)
quite confident	12.73 (7.11)	28.06 (16.84)
slightly confident	17.73 (7.83)	16.22 (8.86)
not confident at all	6.59 (5.3)	3.54 (3.42)


In addition, participants (*n* = 16) reported awareness in 97.66% (*SD* = 3.77) of the control trials, and unawareness in 88.49% (*SD* = 17.32) of the catch the trials. Overall the participants indeed followed the instructions.

### ERP Results

**FIGURE 2:**
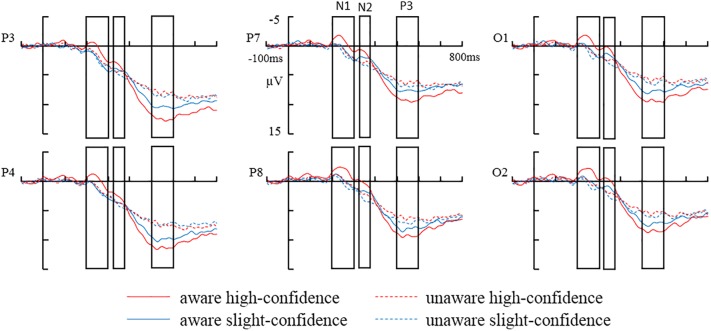
The grand-average ERPs in parietal (P3, P4) posterior temporal (P7, P8), and occipital (Ol, O2) electrodes: aware high-confidence, unaware high-confidence, aware slightly-confidence, and unaware slightly-confidence.

**FIGURE 3:**
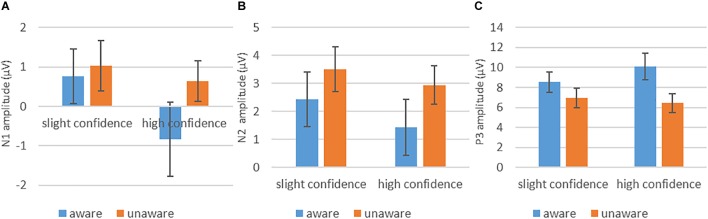
The amplitude for each awareness/confidence condition. **(A)** Nl. **(B)** N2, and **(C)** P3.

The grand-average ERPs in parietal (P3, P4), posterior temporal (P7, P8), and occipital (O1, O2) electrodes are shown in Figure 2. Comparison of the amplitudes of each ERP component across different awareness/confidence conditions is shown in Figure 3.

#### N1

Analysis of variance on average N1 amplitudes showed that main effect of awareness was not significant [*F*_(1,15)_ = 4.18, *p* > 0.05, $\eta_{p}^{2}$ = 0.22]. In addition, the main effect of confidence was significant in the N1 time window [*F*_(1,15)_ = 13.11, *p* < 0.05, $\eta_{p}^{2}$ = 0.47]. The N1 amplitude was more negative in high-confidence trials compared to slight confidence trials (-0.1 ± 0.7 μV vs. 0.9 ± 0.62 μV).

The interaction between awareness and confidence was not significant [*F*_(1,15)_ = 4.07, *p* > 0.05, $\eta_{p}^{2}$ = 0.21] (see Figure 3A). In addition, there was a significant interaction between awareness and areas [*F*_(2,30)_ = 4.1, *p* < 0.05, $\eta_{p}^{2}$ = 0.22]. Simple-effect analysis showed that the N1 amplitude was more negative in aware trials compared to unaware trials over the posterior temporal electrodes (-0.53 ± 0.66 μV vs. 0.42 ± 0.47 μV) [*F*_(1,15)_ = 5.54, *p* < 0.05] and occipital electrodes (-0.54 ± 0.97 μV vs. 0.52 ± 0.65 μV) [*F*_(1,15)_ = 4.71, *p* < 0.05], but not over the parietal electrodes (0.97 ± 0.81 μV vs. 1.57 ± 0.6 μV) [*F*_(1,15)_ = 2.13, *p* > 0.05]. Other interactions involving Awareness were not statistically significant (*p*s > 0.05).

#### N2

Analysis of variance on average N2 amplitudes showed no significant main effect of awareness [*F*_(1,15)_ = 3.93, *p* > 0.05, $\eta_{p}^{2}$ = 0.21]. In addition, the main effect of confidence was not significant in the N2 time window [*F*_(1,15)_ = 3.03, *p* > 0.05, $\eta_{p}^{2}$ = 0.17].

The interaction between awareness and confidence was not significant [*F*_(1,15)_ = 0.47, *p* > 0.05, $\eta_{p}^{2}$ = 0.03] (see Figure 3B). There was a significant interaction between awareness and areas [*F*_(2,30)_ = 5.87, *P* < 0.05, $\eta_{p}^{2}$ = 0.28]. Simple-effect analysis showed that the N2 amplitude was more negative in aware trials compared to unaware trials over the posterior temporal electrodes (1.37 ± 0.82 μV vs. 2.86 ± 0.56 μV) [*F*_(1,15)_ = 4.85, *p* < 0.05] and occipital electrodes (0.96 ± 1.08 μV vs. 2.57 ± 0.79 μV) [*F*_(1,15)_ = 6.29, *p* < 0.05], but not over the parietal electrodes (3.46 ± 1.02 μV vs. 4.23 ± 0.78 μV) [*F*_(1,15)_ = 1.27, *P* > 0.05]. Other interactions involving Awareness were not statistically significant (*p*s > 0.05).

#### P3

Analysis of variance on average P3 amplitudes showed a significant main effect of awareness [*F*_(1,15)_ = 19.65, *p* < 0.01, $\eta_{p}^{2}$ = 0.57]. P3 amplitude was more positive in aware trials compared to unaware trials (9.32 ± 1.15 μV vs. 6.69 ± 0.92 μV), providing evidence for LP. In addition, the main effect of confidence was not significant [*F*_(1,15)_ = 1.19, *p* > 0.05, $\eta_{p}^{2}$ = 0.07]. Most importantly, a significant interaction was found between awareness and confidence [*F*_(1,15)_ = 11.04, *p* < 0.01, $\eta_{p}^{2}$ = 0.42].

Simple-effect analysis showed that for high confidence trials, the difference in P3 amplitude between aware trials and unaware trials reached statistical significance (10.09 ± 1.35 μV vs. 6.44 ± 0.93 μV) [*F*_(1,15)_ = 22.56, *p* < 0.01]. Notably, when the participants indicated slight confidence, there was also a significant difference in P3 amplitude between aware trials and unaware trials (8.55 ± 1.02 μV vs. 6.93 ± 0.97 μV) [*F*_(1,15)_ = 8.63, *p* < 0.05] (see Figure 3C).

Simple-effect analysis also showed that, for aware trials, P3 was more positive in high confident trials compared to slight confident trials (10.09 ± 1.35 μV vs. 8.55 ± 1.02 μV) [*F*_(1,15)_ = 5.58, *p* < 0.05], but, for unaware trials, the different in P3 amplitude between high confident trials and slight confident trials did not reach statistical significance (6.44 ± 0.93 μV vs. 6.93 ± 0.97 μV) [*F*_(1,15)_ = 1.09, *p* > 0.05].

There was also a significant interaction between awareness and areas [*F*_(2,30)_ = 4.56, *P* < 0.05, $\eta_{p}^{2}$ = 0.23]. Simple-effect analysis showed that the P3 amplitude was more positive in aware trials compared to unaware trials over the parietal electrodes (10.98 ± 1.27 μV vs. 7.71 ± 1.06 μV) [*F*_(1,15)_ = 21.41, *p* < 0.01], posterior temporal electrodes (8.67 ± 1.01 μV vs. 6.58 ± 0.79 μV) [*F*_(1,15)_ = 10.36, *p* < 0.01], and occipital electrodes (8.3 ± 1.25 μV vs. 5.77 ± 1.01 μV) [*F*_(1,15)_ = 21.97, *p* < 0.01]. Other interactions involving Awareness were not statistically significant (*p*s > 0.05).

Finally, we have run an additional ANOVA using our entire dataset including data from *all* four confidence level implemented in our study (very confident, quite confident, slightly confident, and not confident at all). Because the participants chose “very confident” and “not confident at all” options infrequently, the data from “very confident” trials and “quite confident” trials were also combined into a high confidence dataset and the data from “slightly confident” trials and “not confident at all” trials were classified as low confident dataset (see Table 1 for proportion of trials in each rating). Consequently ERPs for four types of critical trials were obtained: aware/high-confidence, aware/low-confidence, unaware/high-confidence, and unaware/low-confidence. ANOVA results revealed a significant interaction between awareness and confidence [*F*_(1,15)_ = 12.43, *p* < 0.01, $\eta_{p}^{2}$ = 0.45] and simple effect analysis showed that the positive difference in P3 amplitude between “aware” and “unaware” trials was *not* significant in low confidence level (8.17 ± 1.1 μV vs. 6.85 ± 0.95 μV) [*F*_(1,15)_ = 4.47, *p* > 0.05].

## Discussion

The aim of the current study was to investigate whether P3 reflects visual awareness and/or confidence. For the most part of the discussion, unless we specified, we will discuss results from the data excluding “not confident at all” trials. We found P3 amplitude to be more positive in aware trials compared to unaware trials (evidence for LP). Moreover, we observed a difference in P3 amplitude between aware trials and unaware trials (i.e., LP) for trials responded with slight confidence as well as high confidence.

The main finding of the present study is a positive difference in P3 amplitude between “aware” and “unaware” trials for both confidence levels, indicating a robust effect of awareness on P3 amplitude. Critically, our results differ from that obtained by Eimer and Mazza (2005), who found that P3 amplitude only differed between aware trials and unaware trials when the participants’ confidence was high – not when participants’ confidence was low. They argued that because the effect of awareness was only found in high confidence condition, the effect of awareness found in their experiment must be confounded and contributed entirely by confidence. We argue that it is still possible that the contribution of confidence could not fully explain the difference between aware and unaware trials in their study. Our study shows that indeed the effect of awareness was robust in two levels of confidence ratings and thus suggested that awareness factor alone could module P3.

In fact, the reason Eimer and Mazza (2005) did not find a significant positive difference in P3 amplitude between “aware” and “unaware” trials in low confidence trials might be that participants’ awareness responses were random when they were “not confident at all” (Lamy et al., 2009; Salti et al., 2012). We analyzed separately the entire dataset including data from *all* four confidence levels implemented in our study (very confident, quite confident, slightly confident, and not confident at all). Indeed when the “not confident at all” trials were included there was no significant difference in P3 amplitude between “aware” and “unaware” trials in low confidence trials.

The second piece of our results is that for P3 amplitude, we found significant interaction between awareness (aware vs. unware) and confidence (high vs. slight confidence) even after we removed the data for “not-at-all confident” trials. This suggests that even though P3 is clearly modulated by awareness, confidence might also contribute to the P3 waveform. However, the effect of confidence found here should be interpreted with caution. It is conceivable that given the low level of confidence rating in the “slightly confident” trials, there could be some noise in the awareness response and consequently led to the effect of interaction (smaller effect of awareness in slight confidence, although still significant, compared to that for the high confidence).

In the literature, researchers often argue whether the P3 reflects awareness (Salti et al., 2012) or confidence (Eimer and Mazza, 2005) but never both. Here, we found effects of both awareness (main effect of awareness) and confidence (interaction between awareness and confidence) on P3 amplitude. One might argue that “aware” or “not aware” does not necessarily have to be a binary decision, “confidence” could be related to the extent or magnitude of the awareness (which is still part of the cognitive process for awareness). That P3 amplitude could be modulated by the level of confidence cannot itself prove that P3 does not reflect awareness.

Our results point to the conclusion that awareness modulates P3. But the nature of difference between aware vs. unaware responses remains unanswered. One might argue that in contrast to aware responses, unaware responses could be caused by neural noise which exceeds the signal strength. Fluctuations in attention level at the moment of encoding could also result in sensory signals being amplified on some trials (aware trials) but not others (unaware trials).

There have been some concerns that NCC studies using contrastive experimental designs not only reveal processes directly corresponding to conscious experience, but also other processes that precede conscious perception (i.e., pre-perceptual processes) or follow conscious perception (i.e., post-perceptual processes; Aru et al., 2012; De Graaf et al., 2012; Li et al., 2014; Pitts et al., 2014a; Aru and Bachmann, 2015). Therefore, it is important to note that our results cannot rule out the possibility that the P3 may reflect post-perceptual processes. Previous ERP studies using the inattentional blindness paradigm by presenting words (Schelonka et al., 2017), faces (Shafto and Pitts, 2015), and geometric shapes (Pitts et al., 2012, 2014b) as the critical stimuli showed that P3 correlated with task-relevance, suggesting that P3 reflects post-perceptual processing. For example, P3 might correlate with post-perceptual processing such as working memory (WM). A recent study (Koivisto et al., 2018) manipulated executive WM load which required both maintenance and manipulation of information in WM, and found that executive WM load decreased the amplitude of LP (the positive difference in P3 amplitude between “aware” and “unaware” trials). Their results were in line with the assumption that WM and visual consciousness share resources at a relatively late stage of conscious processing. To some extent, our results are consistent with the idea that P3 is correlated with WM. When participants’ confidence was high, they could easily manipulate contents of WM and produce a response. In such situations, the executive load was low. When the participants’ confidence was low, they could have difficulties in evaluating and manipulating the contents of WM and reaching a decision in the response. In such situation, the executive load was large thus dramatically decreased the amplitude of LP. That is why we found LP was modulated by confidence.

Another main finding of the present study is that VAN is likely an early neural correlates of visual awareness. Our results showed a significant N1 difference between aware and unaware trials over posterior temporal and occipital electrodes. Similarly, there was a significant N2 difference between aware and unaware trials over posterior temporal and occipital electrodes. According previous studies (e.g., Wilenius-emet et al., 2004; Rutiku et al., 2015; Koivisto et al., 2016; Koivisto and Grassini, 2016; Eklund and Wiens, 2018), this significant negative amplitude difference between aware and unaware conditions in N1–N2 latency range over posterior temporal and occipital electrodes is commonly known as VAN. With respect to VAN, our result is somewhat different from previous null results (Lamy et al., 2009; Salti et al., 2012). Koivisto and Grassini (2016) suggested that VAN could not have been found in the previous studies (Lamy et al., 2009; Salti et al., 2012) for three reasons. First, the stimulus was too small to evoke VAN in the previous studies. Second, backward mask paradigm was not sensitive enough to detect VAN. Third, pooling of ipsilateral and contralateral electrodes was not a good way to detect VAN. When Koivisto and Grassini (2016) used larger low contrast stimulus, and ipsilateral and contralateral electrodes were analyzed, respectively, they also found VAN. In present study, we also adopted larger low contrast stimuli. This design may have offered sufficient sensitivity to reveal VAN.

Our finding is in line with previous studies (e.g., Wilenius-emet et al., 2004; Rutiku et al., 2015; Koivisto et al., 2016; Koivisto and Grassini, 2016; Eklund and Wiens, 2018), which suggested that VAN was an early electrophysiological correlate of visual awareness.

As shown in Figure 3A,B, the waveform for the aware/high- confidence condition appeared to be quite different from the other three waveforms which are quite similar. However, we failed to find statistically significant interaction between awareness and confidence most likely due to large variance in the data. Therefore we are less certain about the contribution of confidence in VAN in our current study. Moreover, because NCC studies using contrastive experimental designs may be confounded by processes that precede conscious perception (i.e., pre-perceptual processes) (Aru et al., 2012; De Graaf et al., 2012; Li et al., 2014; Pitts et al., 2014a; Aru and Bachmann, 2015), it is possible that VAN might reflect pre-conscious processes such as attention. Because fluctuations in attention from trial-to-trial could result in sensory signals being amplified on some trials but not others in present study, VAN may reflect fluctuations in attention from trial-to-trial. There are already some relatively early ERP-studies about the relation between visual awareness and spatial attention (Koivisto et al., 2009) or selective attention (Koivisto and Revonsuo, 2008). For example, Koivisto and Revonsuo (2008) found that VAN was influenced by attention. More specifically, the early part of VAN was not completely independent of focused attention, and the later part of VAN was strongly modified by selective feature-based attention. Thus, the further studies are needed to examine the possibility that VAN, which has been assumed to be an early NCC, might actually reflect pre-conscious processing.

## Conclusion

Overall, our results revealed a significant positive difference in P3 amplitude between “aware” and “unaware” trials that was found both in high confidence trials and slight confidence trials. This empirical trend supports the idea that P3 reflects awareness. Moreover, we found a significant interaction between awareness and confidence ratings, suggesting that confidence could modulate P3 as well. Finally, we detected VAN over posterior temporal and occipital electrodes suggesting that VAN over those electrodes is an early neural correlates of visual awareness.

## Ethics Statement

This experiment was approved by the Ethics Committee in Academy of Psychology and Behavior, Tianjin Normal University. All participants gave written informed consent in accordance with the 2013 Declaration of Helsinki and were paid for their attendance.

## Author Contributions

MY, YL, and H-JS designed the experiments. MY and YL prepared the materials and performed the experiments. MY, YL, BS, and H-JS analyzed the data and wrote the manuscript. All authors approved the final version of the manuscript for submission.

## Conflict of Interest Statement

The authors declare that the research was conducted in the absence of any commercial or financial relationships that could be construed as a potential conflict of interest.
